# Connective Tissue Reaction to White and Gray MTA Mixed With Distilled Water or Chlorhexidine in Rats

**Published:** 2009-01-07

**Authors:** Hamid Reza Yavari, Shahriar Shahi, Saeed Rahimi, Sahar Shakouie, Leila Roshangar, Mehran Mesgari Abassi, Sahar Sattari Khavas

**Affiliations:** 1 *Department of Endodontics, Dental School, Tabriz University of Medical Sciences, Tabriz/ Iranian Center for Endodontic Research, Tehran, Iran*; 2 *Department of Histology, Medical School, Tabriz University of Medical Sciences, Tabriz, Iran*; 3 *Member of Drug Applied Research Center, Tabriz University of Medical Sciences, Tabriz, Iran*; 4 *Endodontist, Zanjan, Iran*

**Keywords:** Biocompatibility, Chlorhexidine Gluconate, Mineral Trioxide Aggregate

## Abstract

**INTRODUCTION:** The purpose of this study was to compare the histocompatibility of white (WMTA) and gray (GMTA) mineral trioxide aggregate mixed with 0.12% chlorhexidine (CHX) and distilled water (DW) in subcutaneous connective tissues of rats.

**MATERIALS AND METHODS:** The freshly mixed WMTA and GMTA with CHX or DW were inserted in polyethylene tubes and implanted into dorsal subcutaneous connective tissue of 50 Wistar Albino rats; tissue biopsies were collected and were then examined histologically 7, 15, 30, 60 and 90 days after the implantation procedure. The histology results were scored from 1-4; score 4 was considered as the worst finding. Data were analyzed using one-way ANOVA tests.

**RESULTS:** All experimented materials were tolerated well by the connective tissues after 90-day evaluation, except for the WMTA/CHX group that had significantly more mean inflammatory scores (P<0.001). There was a statistically significant difference in the mean inflammation grades between experimental groups in each interval (P<0.001). After 90 days, GMTA/CHX group had the lowest inflammatory score.

**CONCLUSION:** Although adding CHX to WMTA produces significantly higher inflammatory response, it seems a suitable substitute for DW in combination with GMTA. Further research is necessary to recommend this mixture for clinical use.

## INTRODUCTION

Mineral trioxide aggregate (MTA) has become the material of choice for several clinical applications. It is an effective material for pulp capping (1), sealing and repairing root perforations (2-4) and to create an apical barrier in teeth with open apices (5,6). Studies have shown that MTA is suitable root-end filling (7) and i*n vitro *and *in vivo* papers have reported that MTA is a biocompatible material. It has been shown to promote osteoblastic activity (8,9) and was less cytotoxic than amalgam, IRM or super EBA (10). MTA has antibacterial activity (11), minimal toxicity and pulp irritation, and mild periapical inflammation (2, 8). Furthermore, MTA increases the levels of alkaline phosphatase and osteocalcin as well as interleukin production (IL6, IL8), periodontal ligament attachment, cementogenesis, and dentinal bridge formation (2,8,9,12-14).

The major components of MTA are Portland cement (75%), bismuth oxide (20%) and gypsum (5%) (15). It also contains trace amounts of MgO, K_2_SO_4_ and Na_2_SO_4_. The major components of Portland cement are a mixture of dicalcium silicate, tricalcium silicate, tricalcium aluminate, and tetracalcium aluminoferrite (15). MTA is prepared as a mixture of powder and water and is used in a slurry form, which gradually hardens in the oral environment (5).

In 2002, white MTA (WMTA) (tooth-colored formula) became available. The new formulation is iron free unlike gray MTA (GMTA) and therefore does not stain teeth (13). Holland *et al.* have investigated the biocompatibility of the WMTA in rat subcutaneous tissue and have found similar results with those reported for the GMTA formulation (13).

Chlorhexidine (CHX) is an antimicrobial agent belonging to a group of N5 derivatives of 1,6 bis- biguanidohexane (16). It has been widely used as an antiseptic and is active against gram-positive and gram-negative bacteria, facultative anaerobes and aerobes, moulds, yeasts and viruses (17). It acts by adhering to cell wall of the microorganisms and causing leakage of intracellular components and eventually leading to cell death (16,17).

In endodontics, CHX has been found to be an effective antimicrobial agent when used as a root canal irrigant (17). Stowe *et al.* have shown that substitution of 0.12% CHX gluconate with sterile water in tooth colored Pro-Root MTA enhanced the antimicrobial effect of this material *in vitro *(11). However, Hernandez *et al.* showed that this mixture induced apoptosis of macrophages and fibroblasts *in vitro *(18)*;* however there are as yet no *in vivo* studies to evaluate its cytotoxicity.

The aim of this *in vivo* study was to compare the histocompatibility of WMTA and GMTA when mixed with CHX 0.12% or distilled water (DW), in subcutaneous connective tissue of rats.

## MATERIALS AND METHODS

Fifty male, 5-6 months old Wistar Albino rats weighting 270±20 g were used in this study. The criteria of the Helsinki Declaration regarding laboratory animals were considered in all steps of the project (19). The study has been approved by Ethics Committee of Tabriz University of Medical Science.

The following materials were investigated:

**Group 1:** WMTA (Angelus, Londrina, Brazil) mixed with DW

**Group 2:** GMTA (Angelus, Londrina, Brazil) mixed with DW

**Group 3:** WMTA Angelus/CHX 0.12% 

**Group 4:** GMTA Angelus/CHX 0.12% 

**Group 5:** Control group (empty tubes)

Freshly mixed test materials were placed in clean, sterile, polyethylene tubes (Eastern Medikit LTD, Gurgaon, India) with 1.1 mm inner diameter and 0.8 mm length.

MTA was mixed with distilled water according to manufacturer’s recommendation and was applied with a plastic carrier. For groups 3 and 4 CHX 0.12% was substituted with DW. Fifty empty polyethylene tubes served as controls.

The dorsal skins of animals were shaved under general anesthesia using diethyl ether (Parchemie, Tehran, Iran) and anesthetic chamber technique and disinfected with 5% iodine solution. Five incisions were made on the backs of the albino rats; incisions were made over a length of 1 cm using no.15 blade in a head-to-tail alignment. The skin was reflected, and the implantation materials were inserted into spaces created by blunt dissection. To prevent interactions of materials, the tubes were placed at least 2 cm far from each other (2 tubes in one side of the rats back and 3 in the other). The evaluations were made 7, 15, 30, 60 and 90 days after surgical implantation (20,21).

In each examination period, 10 animals were euthanized by administrating high doses of anesthetics. The dorsal skin was shaved, and the tubes were excised together with the surrounding connective tissues. The specimens were kept in a 10% formalin solution for two weeks (Merk, Darmstadt, Germany) until histological processing. Sections with 5µm thickness were taken from specimens and placed in paraffin blocks and stained with hematoxylin and eosin. Evaluations were made in a light microscope (Leitz, Oberkochen, Germany) at ×400 and ×800 magnifications. Quantitative evaluations of inflammatory cells (lymphocytes, plasmocytes, polymorpho- nuclear leukocytes [PMN], macrophages, and giant cells) were made in ten separate areas of sections at ×400 magnifications. An average value for each material was obtained from the sum of cells counted in ten separate areas (22,23).

Reactions were scored and evaluated as: 

Score 1: few inflammatory cells without edema

Score 2: <25 inflammatory cells, wavy collagen fibrils deposition and fibrosis

Score 3: 25-125 inflammatory cells, edema and vascular congestion

Score 4: >125 inflammatory cells, edema and vascular congestion and fibrin deposition (20,21).

Statistical analysis was carried out using one-way ANOVA. To determine differences between groups, LSD test was performed. Statistical significance was defined as P<0.05.

## RESULTS

**Figure 1:**
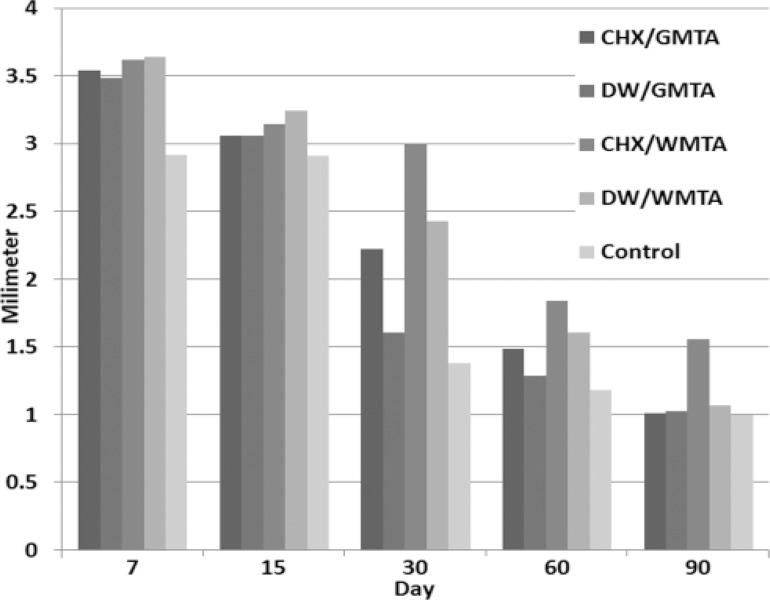
Mean inflammation in experimental and control groups in different intervals

**Figure 2:**
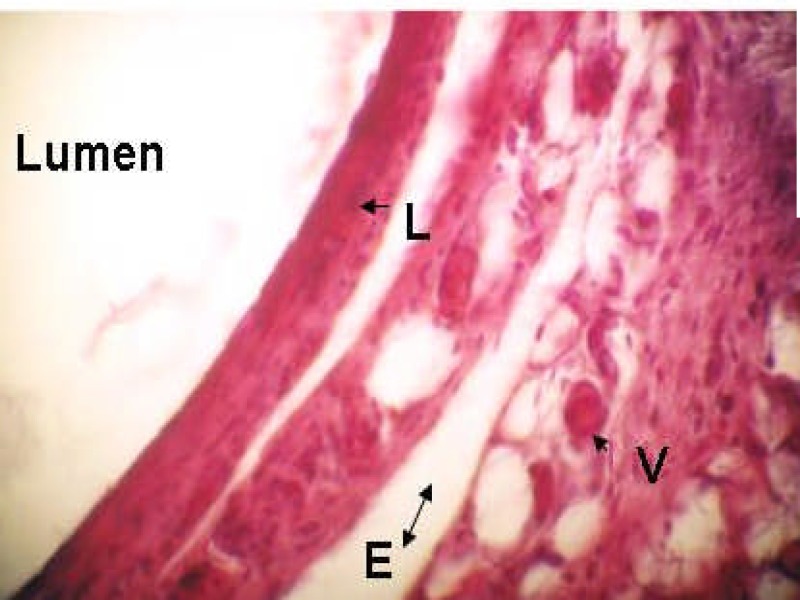
The 30-day old CHX/GMTA specimen with grade II inflammation. V:venule L: Lymphocytes E:Edema (×400 Mag)

Histologic findings are presented in Table 1. There was a statistically significant difference in the mean inflammation scores among groups in each interval (P<0.001) (Figure 1), (Table 2). In 7-day and 15-day specimens there was no statically significant difference between the mean inflammation scores of all groups. 30-day specimens (Figure 2) showed statistically significant difference between the mean inflammation scores of all groups (P<0.001). In 60-day specimens WMTA/CHX showed the highest mean inflammation score, however, there was no statistically significant difference between the mean inflammation scores of the others (P<0.05). In 90-day specimens WMTA/CHX showed the highest mean inflammation score and there was no statistically significant difference between the mean inflammation scores of the other groups (P<0.05).

## DISCUSSION

Although test materials were directly applied subcutaneously in some studies (24), the implantation of the materials in tubes is advocated in others (4,22,23,25-27); in these cases silicon (22,23), polyethylene (25,28), teflon (29), or dentin tubes (4) have been utilized. Applying the test materials in tubes simulates the clinical conditions (28). When compared with the direct application of the material, this method helps to provide stabilization of the material placement and to achieve the standardization of the material-tissue interfaces (28). In this study, polyethylene tubes with 1.1 mm inner diameter were used.

The reactions to empty tubes in this study were similar to others (25,28,30), who found polyethylene tubes caused few or no reactions in subcutaneous connective tissues. Researchers reported that there were some inflammation around the tubes until the end of second week, and this inflammatory infiltration subsided after the third week (25,30). This reaction was the result of the trauma produced during the placement of tubes (30).In the present study MTA-Angelus manufactured in Brazil was chosen because it presents a similar composition to ProRoot MTA according to the manufacturer. Duarte *et al.* have demonstrated that both materials released calcium and provide alkaline environment. Moreover, when used in direct pulp capping or pulpotomy, both materials were biocompatible and effective to produce complete pulp healing (31). Menezes *et al.* also showed that the tissue reactions were identical for ProRoot MTA and MTA-Angelus (30). Xavier *et al.* showed that MTA-Angelus presented the best marginal adaptation in comparison with super EBA and vitremer (32). The toxic effects of white or GMTA in mixture with CHX 0.12% or DW were examined at 7, 15, 30, 60 and 90 days (20,21). When assessing the biocompatibility of a material, the delayed detrimental effects were considered to be more important that its initial effects (28). Seven-day results of both materials showed that moderate inflammatory response developed in subcutaneous connective tissues of rats, but these reactions subsided by the 60^th^ day and were further reduced on the 90^th^ day.

**Table 1:** Distribution of inflammation grades (IG) percentage in groups in five intervals

**Groups **	**IG**	**Intervals (Day)**				
		**7**	**15**	**30**	**60**	**90**
WMTA/DW	I	0	0	0	42	93
	II	3	0	57	55	7
	III	30	51	43	3	0
	IV	67	49	0	0	0
GMTA/DW	I	0	0	39	72	97
	II	0	0	61	28	3
	III	48	54	0	0	0
	IV	52	46	0	0	0
WMTA/CHX	I	0	0	0	16	54
	II	0	5	0	84	36
	III	38	76	100	0	0
	IV	62	19	0	0	0
GMTA/CHX	I	0	0	0	51	99
	II	0	0	78	49	1
	III	46	94	22	0	0
	IV	54	6	0	0	0
Control	I	0	0	63	82	100
	II	8	9	36	18	0
	III	92	91	1	0	0
	IV	0	0	0	0	0

There are further studies that support these findings demonstrating fibrous connective tissue formation around MTA and amalgam (28); which indicates that these materials are well tolerated by tissues. Interestingly we showed that MTA (white or gray) in combination with 

**Table 2:** Result of statistically analysis between groups in 5 intervals.(mean inflammation grades± standard deviation)

** Interval** **Groups **	**7-day**	**15-day**	**30-day**	**60-day**	**90-day**	**P value**
WMTA/DW	3.54±0.50	3.06±0.23	2.22±0.41	1.49±0.50	1.01±0.10	<0.001
GMTA/DW	3.48±0.50	3.06±0.67	1.61±0.49	1.29±0.47	1.03±0.17	<0.001
WMTA/CHX	3.62±0.48	3.14±0.47	3 ±0.00	1.84±0.36	1.56±0.67	<0.001
WMTA/CHX	3.64±0.54	3.24±0.43	2.43±0.49	1.61±0.54	1.07±0.25	<0.001
Control	2.92±0.28	2.91±0.28	1.18±0.38	1.38±0.38	1±0.00	<0.001

CHX or DW, could decrease the mean inflammatory response significantly from 7 to 15 days, while the empty tubes (control group) could not. This adds further weight to the argument that MTA may be used as a biocompatible material with CHX and DW to reduce inflammatory response particularly in early tissue contact.

Moretten *et al.* examined the biocompatibility of MTA by subcutaneous and intra-osseous implantation (33). MTA initially elicited severe reactions with coagulation necrosis and dystrophic calcification; the reactions however subsided gradually to a moderate level. The subcutaneous implantation results of our study concur with Moretten *et al.* (33).

The mean inflammatory score reported in our study differ from those of Yaltirik *et al.* (28); this may be due to differences in scoring microscopic evaluations, which ranged from 0 to 3 in Yaltirik *et al.*'s study (28) (1 to 4 in our study). In addition we reported the mean inflammatory scores obtained from the sum of cells which were counted in 10 separate areas. The average value was not rounded and was precisely reported, which is more accurate than the overall mean value. This may be why our results showed significant difference in 15, 30 and 60 days.

Tissue reaction to empty tubes in all intervals was milder than other experimental groups according to our results. On the 60^th^ day no significant difference were present between control and GMTA/DW group; none of the studied groups except for WMTA/CHX showed statistically significant difference in inflammatory response in 90 days. This is consistent with the findings of Yaltirik *et al.* (28).

Our findings showed that white and gray MTA mixed with DW had significantly different tissue reactions in all intervals except for the 90-day interval. This can be the result of different chemical properties of the two materials. Asgary *et al.* showed that the major disparity is FeO, compound being omitted from the WMTA formulation (34). It also has more bismuth oxide than GMTA (34). Yamamoto *et al.* demonstrated that this oxide can cause toxic effects and have negative effects of cell growth (35). Perez *et al.* showed that osteoblasts are more sensitive to WMTA rather than GMTA and cells attached to WMTA were not viable (36). Furthermore, the various tissue reactions of these two materials may be due to the difference in their surface roughness and topographies (36). Matt *et al.* also showed that GMTA had more sealing ability than WMTA to be used as a root end filling material (37).

The mixture of MTA/CHX has been studied in previous *in vitro* studies (11,18). Stowe *et al.* showed that CHX improved antibacterial activity of MTA (11). Hernandez *et al.* (18) showed that WMTA could induce apoptosis of macrophages and fibroblasts *in-vitro*; however *in vitro* studies are fundamentally different from *in vivo* ones, as proteins, tissue fluid and other factors can reduce the toxic effects of materials (16). Sauthard *et al.* have demonstrated biocompatibility of CHX (38). Other researchers have reported similar results (39-41), but it is not known why this material acts differently when mixed with GMTA and WMTA. In our study CHX showed good biocompatibility with GMTA but not with WMTA; this may be due to the physical and chemical properties of the materials, yet the precise reasons are still obscure.

## CONCLUSION

According to results of this *in vivo* study, CHX can be a good substitute for DW in mixture with GMTA; however, WMTA elicits more inflammatory response in combination with CHX. CHX has no negative effects on MTA-dentin bonding (42); more studies about physical and chemical properties of MTA/CHX mixture are needed.
